# Writing good economics: How texts ‘on the move’ perform the lab and discipline of experimental economics

**DOI:** 10.1177/03063127221079600

**Published:** 2022-04-03

**Authors:** Kristin Asdal, Béatrice Cointe

**Affiliations:** 1 University of Oslo, Oslo, Norway

**Keywords:** economics, experiments, texts, performativity, laboratory

## Abstract

How is objectivity accomplished in laboratory economic experiments? To address this question, this paper focuses on a modest and mundane thing: the written instructions that guide experimental subjects in the lab. In a material-semiotic perspective, these instructions can be understood as text-devices. We follow text-devices ‘on the move’ from their very writing, through the lab, the review process and out into the journal article. To do so, we analyse ‘text-author ensembles’, which are journal articles together with practice-oriented interviews with their authors. We show that instructions act not simply as texts, but as experimental instruments that also perform the procedure of experimental economics. They draw together the procedural, material and rhetorical dimensions of experimental work in economics, and link the lab setting to collective validation procedures within the discipline of economics. To achieve this, experimental economists rely on qualitative writing skills refined in collective writing and reviewing practices. These text-devices ‘on the move’ alert us not only to the role of writing and writing skills in the production of scientific knowledge, but to the role of texts as material and semiotic objects that can produce facts as well as labs and disciplines, and that are key to the accomplishment of objectivity in experimental economics.

The past few decades have seen the rise of experimental techniques in economics. Both lab and field experiments are now widespread in economics, where they are used for a range of purposes: for example, to test theory, to characterize economic subjects, to inform and perform policy (Guala, 2007; Mirowski and Nik-Kah, 2007; Muniesa and Callon, 2007), or to elicit monetary valuations for things for which no markets exist (Asdal and Cointe, 2021; Schmidt, 2021; Teil and Muniesa, 2006). It is, however, only quite recently that experiments in economics gained ground, and this is partly why they are especially interesting to study. For most of its history, economics worked without experimenting, and it is intriguing how experimental practices have been incorporated into model-based knowledge production practices. This paper takes such experiments – more precisely, laboratory experiments – as the starting point for exploring this knowledge production process and, related, the accomplishment of objectivity in economics.

There is already a wealth of studies on how ‘economists work and think’ (Morgan, 2012). These studies span a wide range of approaches, including the history of economic thought (Backhouse and Cherrier, 2017), the philosophy, epistemology and methodology of economics (Guala, 2005; Mirowski, 1989, 2001), and its sociological and institutional organization (Fourcade, 2009). Economics has also been studied within the field of Science and Technology Studies (STS), then often with a focus on economics in its relations to the economy. One of the contributions of STS to the study of economics has been to highlight ‘performance’ and ‘performativity’ as crucial notions to understand how economics relates to its object. Economic knowledge, it is argued, does not so much represent the world as it provokes and performs its worlds (MacKenzie et al., 2007; Mitchell, 2005; Muniesa, 2014). This approach has been linked to the analysis of two kinds of objects. On the one hand, research on market devices (Callon et al., 2007) and on the interactions between the making of economic facts and the organization of economic life (MacKenzie, 2003) has shown how economic knowledge equips markets and market agencies, and thereby performs the economy. On the other hand, studies of economics-in-the-making have investigated how economic models and experiments perform economic worlds in order to study them (Guala, 2007; Mitchell, 2005; Morgan, 2012; Schmidt, 2021; Yonay and Breslau, 2006).

It is thus widely accepted that economics is both ‘performed’ and ‘performative’. What has received less attention is how specific modes of performing are made to count as economics. This relates directly to an issue that research on the making of economics has, so far, somewhat neglected, namely the conditions of possibility, through provoked behaviours and performed agencies, for collectively validated knowledge.^1^ This is the research problem we seek to answer. In doing this, we pursue the related issue of objectivity in the social sciences (Asdal and Hobæk, 2020; Weber, 2012).

Historical and sociological analyses of objectivity have demonstrated how objectivity is anchored in concrete, collectively validated practices and devices: images and atlases (Daston and Galison, 2007), numbers and quantification procedures (Porter, 1995), models and narratives (Breslau and Yonay, 1999; Morgan, 2012). What are the practices and devices accomplishing objectivity in experimental economics? And can a further understanding of the knowledge production in laboratory experimental economics tell us something about how objectivity is done differently here than in other arenas and disciplines? Can an analysis of the production of objectivity in experimental economics add to our understanding of the objectivity question in the sciences more broadly?

The argument of this paper is that the objectivity of economic experiments is in part accomplished by a seemingly modest and quite mundane ‘text-device’, and that this device enables procedural, material and rhetorical elements to work together towards experimental results. This text-device is the experimental instructions. These instructions are not simply texts, but rather form an experimental instrument. In exploring this particular instrument ‘on the move’ (Asdal and Jordheim, 2018) through the writing, experimenting, reviewing and publishing process, we are alerted not only to the role of writing and writing skills in the production of scientific knowledge, but also to the role of texts as material *and* semiotic objects that work to produce facts as well as labs and disciplines.

## Following a text-device ‘on the move’: The instructions

The physical setup of economic laboratories is not particularly distinctive. Like many psychology or marketing experiments, economic experiments take place in classrooms divided into individual, isolated booths where subjects perform tasks on a computer. Yet the interactions that occur in the lab, and the results that are extracted from it, are quite distinctive, as ethnographies of economic laboratories have shown (Böhme, 2016; Sorgner, 2017). This means that the physical setup and instrumentation alone tell us relatively little of what is going on in the lab.

However, there is another, less visible device involved which can help us make sense of these labs: the written instructions that guide participants through an experiment. These instructions, we argue, are text-devices where much of the work of experimental economics happens. They need to be considered if we are to understand what makes economic experiments compelling performances, and thus how experimental economics produce economic knowledge. Indeed, we find the experimental instructions to be a crucial part of the design of economic experiments, of the performance of economic situations and behaviours in the lab, and of the collective validation and circulation of economic experiments and of their results. In other words, they not only make up the labs, but also shape the procedures of experimental economics. To understand how they do so, we need to be attentive to their double function as narratives of the experiments and as material entities – documents that may be moved around throughout the research and publishing process.

The centrality of instruction texts at every step in the making of an experimental economic result prompts us, as science studies scholars, to re-interrogate the role of texts in the production of scientific knowledge. To analyse what these instruction texts do, we need to bring together, but also move beyond, studies of the role of texts in the making of both economic and experimental knowledge.

Studies of economics have addressed texts – especially in their narrative and literary construction – as a way to work with models. For example, based on the close reading of economic texts from different periods, Morgan (2012) argues that narratives are key instruments to link the ‘world in the model’ and ‘the world that the model represents’, that is, to link economic models and economic reality. From this observation, she makes the compelling argument that these narratives form an epistemic practice, not simply a rhetorical one (Morgan, 2012: 239). Morgan is primarily interested in meaning and content, not in the texts themselves as material-semiotic entities. However, her argument can be extended to show that stories and texts in economics are not mere accounts but play an active role in fact-making.

Breslau and Yonay (1999) suggest as much. Adopting a practice-oriented approach informed by laboratory ethnographies, they analyse the writing of articles as central to the doing of model-based economics. In economics based on mathematical modelling, they argue, scientific work is ‘dedicated to producing the orderly account found in the report’ (Breslau and Yonay, 1999: 329), and it is the very format of the paper that produces the ‘clear and compelling performance’ (Breslau and Yonay, 1999: 322) of the model. In this format, the setup of the model by the economists and the performance of the model are presented in distinct sections, thereby separating the agency of the economists from the agency of the model. The whole modelling process is shaped by the aim of writing an account that presents the model as external to the text. As such, it can be likened to laboratory science.

Models are not the centrepiece of experimental economics. Economic experiments usually refer to a model, but it is not the main instrument in the research process. The experimental setup is, and this setup is largely established by writing the instructions. This implies that an analysis of experimental economics cannot consider texts only as the endpoints of research, as final accounts in the form of journal articles or books. It also needs to attend to the role of texts as experimental devices.

This, in turn, brings us back to classic laboratory studies on the role of texts and inscriptions in the making of experimental facts (Callon et al., 1986; Latour, 1995; Latour and Bastide, 1986; Law, 1986). The notions of inscriptions and inscription devices specifically address the material-semiotic aspects of lab science. They explain how objects are made moveable by being abstracted and inscribed into computers, graphs, diagrams or tables. When analysing inscriptions, the focus is on how natural or experimental objects are translated into paper. Yet there is an important difference in our case: While crucial to the experimental process, instructions are not inscriptions, but instruments. They are written *before* the experiment takes place and are not designed to translate objects out of the lab, but to make them move in the lab (they do other things too, but let us start here, from this basic requirement); they are later appended to the final article to support it, moving along with its claims and findings. This could liken them to protocols (Lynch, 2003), but whereas protocols serve as guides or recipes, instructions are part of the experiments.

We do not focus on texts in opposition or in contrast to the material, nor in a way that would overlook the materiality of the laboratory setting of experimental economics. The very notion of text-device that we propose underscores this. If we follow the text-device as it moves between the lab setting and the discipline of economics as an institution, we can analyse how texts and written documents participate in economic practices both in the lab and in the discipline.

This needs to be specified further, as it is precisely how the text-device is set ‘on the move’, how it is made to move between different sites and operate at these different sites, that enables it to perform. In order to grasp this, we need to shift from ‘narratives’ to ‘texts’, as this enables us to approach experimental economics from a material-semiotic perspective and to ask: How are texts concretely and practically involved in experimental economics work? In asking this question, we are interested in the texts not just as repositories of meanings, but also as concrete objects that move and do things.^2^

Empirically and analytically then, our investigation is guided by an apparently simple question: How do these instruction texts move? How do they connect the labs and the discipline of economics, and contribute to making economic experiments into compelling performances? By following these text-devices, we hope to clarify how rhetorical and material elements work together in a quite procedural manner to produce objectivity in experimental economics. Hence, we follow how experimental economics proceeds.

## Our method: Following ‘text-author ensembles’

To follow the instruction texts as they move in and out of the labs, our method was to analyse economic papers together with interviews with their authors, that is, ‘text-author ensembles’ (see also Asdal and Cointe, 2021). Concretely, our material consists in a series of interviews with economists who specialize in experimental work. We have not selected one particular area of economics, being open to the diversity of uses of experiments in the field. To contact interviewees, we followed suggestions from previous interviewees and identified relevant research groups using the list of experimental economics labs compiled by the *Laboratoire d’Economie Expérimentale de Montpellier*.^3^ For practical reasons, we limited ourselves to economists based in Norway and France and publishing predominantly in English. The nine experimental economists we interviewed are academic researchers: They work in academic institutions and their main job is to produce scientific papers.

Our objective was not to strive for an extensive mapping of the use of experiments in economics, but rather to collect in-depth accounts of experimental work. Thus, we relied on a ‘practice-oriented interviewing method’ (Asdal and Reinertsen, 2022; Mangset and Asdal, 2019). For this, we asked each interviewee to provide one of their papers ahead of the interviews, and together we retraced the making of this paper – including the conception of the experiment, the writing process and the publication. Three interviewees were co-authors and talked about the same paper. This provided us with a collection of papers on topics as diverse as corporate social responsibility, innovation, management of common resources, trust, nutritional and energy labelling, and political corruption in elections, relying mostly on lab experiments – one paper/interview was based on field experiments. For the purpose of this article, we focus on the interviews and papers using lab experiments.

In addition, we rely on another set of thirteen interviews collected beforehand to investigate experimental methods in market research (Asdal and Cointe, 2021). These thirteen interviews are not central to the analysis presented in this paper, since they were not limited to economists, but they helped us grasp the differences in experimental practices between economics and other fields.

Unless stated otherwise, the descriptions and accounts presented here emerge from the interviews, cross-analysed with the papers. Our approach is similar to that previously used to study note-writing in bureaucracy, in that we consider interviewees’ accounts not as providing the ‘truth’, but ‘as accounts of what is perceived as legitimate in [a] professional group’ (Mangset and Asdal, 2019: 9). Our method based on ‘text-author ensembles’ enabled us to extract practices that are not easily observable, such as writing, coding, performing statistical analyses or valuing results, and from there to analyse these practices. We cross-checked and complemented our interview-based descriptions with previous ethnographies of economic laboratories (Böhme, 2016; Muniesa and Callon, 2007; Sorgner, 2017; Teil and Muniesa, 2006) and with works on the methodology of experimental economics written by economists (Bardsley et al., 2010; Kagel and Roth, 1995) and non-economists (Guala, 2005, 2007; Morgan, 2012). One of the authors also acted as a subject in an experiment.^4^

## Introducing economic experiments: The textbook version

What exactly is an economic experiment? The laboratories of economics are quite mundane: They are rooms lined with small cubicles, each equipped with a computer. People taking part in economic experiments – economists call them ‘subjects’ – are invited to sit in the cubicles, in which they are unable to see other subjects. They can ask the experimenters for help, but cannot communicate with fellow participants. Thus isolated, they play ‘games’ with money at stake. Concretely, they make decisions on the computer. Their choices determine how much money they will earn at the end of the experiment.

The games we encountered in our study mostly revolved around very abstract situations, with no explicit connection to everyday life.^5^ As some of our interviewees noted, not all economic experiments have to be abstract, but we focus on the abstract kind, on which we have more material and for which, arguably, the specificities of economic labs are more salient. Experimental sessions typically last for one to one-and-a-half hour, at the end of which participants check out with someone from the lab who gives them their earnings. Unless the research question requires a specific type of person to take part, ‘subjects’ are often university students; they can be from any discipline, though experimenters have their preferences, such as avoiding economics or psychology students or favouring science students.^6^

Experimental setups in economics are quite codified. Other scholars have shown how they isolate the laboratory (Sorgner, 2017), frame specific interactions (Böhme, 2016), and create spaces in which economic norms apply (Guala, 2007). Interestingly, similarities in practices and setups seem to unite experimental economics more than their findings or relationships to economic theory. As Guala’s distinction between ‘testers’ and ‘builders’ shows, there are different conceptions of what experiments can bring to economic theory: They can be used to test and challenge it, or to engineer situations in which it applies (Guala, 2007). However, there are shared norms and practices regarding how to carry out economic experiments. It is those shared norms and practices that we refer to as the ‘textbook version’ of experimental economics.

Our use of the term ‘textbook’ does not imply that there is one textbook defining the methodological and practical rules of experimental economics, but should rather be understood in the sense of typical, classic version. The experimenters we interviewed gave remarkably consistent descriptions of the norms and methods they followed, suggesting that there is a well-established, ‘textbook’ way of doing experimental economics. Indeed, one sternly explained, ‘there are certain norms that you abide by. So, if you don’t abide by these norms, then you don’t get published in economic journals’ (Experimenter 4).

Some of these norms can be traced back to theoretical and methodological texts. Guala (2005, 2007) identifies a series of articles and manuals from the late 1970s–80s as foundational to the discipline, among which Vernon Smith’s induced value theory and its ‘five precepts’ of experimental economics (which notably codified the use of incentives) (Smith, 1976, 1982). But what we call the ‘textbook version’ also encompasses concrete, practical aspects of experiments that are not necessarily discussed in manuals or articles, but that were explicated in our interviews.^7^

One core rule is the use of monetary incentives. This rule is widely discussed in the methodological literature (Smith, 1976; see also, e.g. Bardsley et al., 2010; Guala, 2005). Interviewees emphasized it unequivocally:

So what are these norms, first and foremost, we incentivise financially. So that … subjects are paid according to their performance in the game. (Experimenter 4)

Economists do not believe what people say, but they believe what people do. For them, the market reveals [preferences]. … If I make a decision in the laboratory, it will have consequences. In laboratory experiments such as the prisoner’s dilemma, … the monetary reward that participants get in the end will depend on the decisions they made. (Experimenter 8)

Another major rule that stands out in the interviews is what one experimenter referred to as ‘very strong norms against deception’ (Experimenter 4). Economists are not supposed to lie to their subjects. They do not have to tell them everything about the experiment, but ‘everything [they] say is true’ (Experimenter 4). Our interviewees stress that experiments involving some degree of deception or misleading information are extremely hard to publish in economics journals – several shared stories about papers that they or some colleagues struggled to publish for this reason (Experimenters 3, 8, 9). As one explains, ‘When you want to publish in economics journals, if they see that you have lied to subjects, you cannot be published’ (Experimenter 8). Another concurs: ‘For instance, if you tell them, “you are going to play with another participant”, and in reality, they are going to play with a computer: Never publishable in economics’ (Experimenter 5).

These two rules are in sharp contrast with experimental practices in other social sciences, and especially in psychology. Psychologists only pay a flat compensation for their subjects’ time, not an incentive, and they usually lie about the actual purpose of the experiment.^8^ This contrast with psychology largely owes to the different norms and interests of the two fields. In psychology experiments, telling subjects the purpose of the experiment will often destroy the very effect under study. All the same, our interviewees often stressed it as a major difference.

There are also norms and standards of practice to ensure experimental control and enable experimenters to identify causal relations. For instance, experimenters seek to enforce *ceteris paribus* conditions, in which no uncontrollable parameter may influence the results. They also try to avoid what economists call ‘confounding’ (when players and experimenters have a different understanding of the game) and ‘demand effects’ (when players alter their behaviours to try to please the experimenters). Experimenter 4 explains:

We’re very careful not to say anything about the purpose of the study. And we’re also very careful to make sure that they understand that they cannot be observed by others, and there’s perfect anonymity. So this is in order to avoid what we call demand effects. So if I told them, I’m doing this study hoping to find such an effect, then, maybe some of them would please me, or some of them would be afraid that if they didn’t do what we expect, they wouldn’t be invited again. (Experimenter 4)

Last, some practical elements contribute to the similarities across economic experiments. For example, in the literature, ‘there are some standard games’, ‘some games that are commonly used’ (Experimenter 2). When designing experiments, economists will often pick a game from this set of standard games and adapt it to their research questions. For instance, the ‘dictator game’ is used to measure altruism, and ‘public good games’ are used to study cooperation.

Further, economic experimenters now often use the same software to programme games on the computer, z-Tree. For the most common games, ready-made programmes are even available for download.^9^ Several interviewees referred to it, and z-Tree is also used in the research centres studied by Böhme (2016) and Sorgner (2017). Not just the physical settings of the labs, but the very computer interfaces on which the games are played are thus very similar across experiments.

Coming out clearly from the interviews, then, and in line with previous research, is that the setups of economic experiments are quite standardized, in terms of both methodological rules and practical arrangements. Among the key elements of these setups, we find an apparently modest piece of text: The instructions.

## Writing economic experiments

### Producing the experimental instrument: Writing good instructions

After sitting at their computers, participants in an economic experiment are given written instructions. Usually, these are handed on a sheet of paper, but they can also be displayed on the computer screen or using a projector. The text is then read aloud for everyone in the room to hear. Addressing participants in the second person, it details everything they need to know to take part in the experiment and explains what will happen, step by step. The instructions work together with the computer programme: They prepare the participants for what will show on the screen, and prompt them to click buttons or fill in boxes. For example:

To answer question B, you must fill in thirteen numbers in a table. … You must write one answer for every line in the table. You can write any integer you like between 0 and 60. … To be sure that the instructions are clear enough, we ask you again to answer some questions which will appear on the screen. You will get a couple of minutes to read through the instructions on your own now, and to answer the questions. … When all have completed the questions, and have clicked the ‘Continue’-button, the experiment will continue. (Experiment A).

The experimenters devote much care and time to writing these instructions. In fact, it is a major part of their work.

One of the first things that we really work hard on, is to write down the design and the detailed instructions … – we work *a lot* with the detailed instructions, and every single word in the instructions needs to be well thought through to avoid any misunderstandings … (Experimenter 1)

There is good reason for all this attention, as Experimenter 4 underlines: ‘We do a lot of work with the instructions, because *that’s really our instrument*, right. Together with the screenshots [of the computer interface]’ (emphasis added).

After the experimenters have formulated hypotheses and decided upon the structure of the experiment, writing the instructions is a key part of turning the conceptual idea into a practical experimental design. It happens alongside the programming of the software on which the game is to be played, in a back-and-forth process, confirming that the two work together to set up the lab. Experimenters are especially careful to ensure that what participants are told to do (by the instructions) aligns with what they do (on the computer).

We start writing instructions, and programming, and then, so go back and forth between those, because we understand that, ‘oh, we forgot to think about x or y’, and we have to update the instructions, we write the instructions, and then we find out ‘oh, we haven’t been clear about this!’ we have to update the programme. (Experimenter 2)

This iterative writing-and-programming also involves checking the literature and carrying out pilot runs in the lab, very often resulting in adjustments to the experimental design, in a ‘back-and-forth process that *may* take several months’ (Experimenter 1):

So if the experiments were due in mid-October, for example, we would typically try to finish a first draft of the instructions in … Late August. And then, discuss them among us, think through it once again, try to do the programming, and then, we would typically come up with some adjustments that we need to make. And then, we need to … Do test runs in the lab to see if everything is working, which is – it usually is not the first time. (Experimenter 1)

[This is] not a very structured process. And then, once we start to write the programme and write the instructions, then things get more concrete, and as you get more concrete, new problems pop up, and we have to have a new meeting to discuss … Is this the instructions we want? We have done it this way now, is that the best way of doing it? (Experimenter 3)

### How the instructions proceed: Enacting the rules of the game, the design of the experiment and the lab

What makes the instructions so pivotal and hence worthy of so much time and attention? Experimenters inscribe three things in the same text: the rules of the games played in the experiment, the design of the experiment, and the laws of the lab. Taken together, these constitute a procedural setup connecting what goes on in the lab to the experimenters’ research questions and to economics as a discipline. The instructions constitute the procedure that is a condition of possibility for compelling economic experiments. Let us stay with this notion of procedure, as it is instructive for our take on experimental economics. ‘Procedure’ quite literally means steps taken, an act performed or the act or manner of proceeding – that is, moving forward. Interestingly then, the notion combines the act of prescribing action and the way actions are part of a forward-oriented movement. Thus, in other words, actions are ‘on the move’ in an ordered manner. The element that allows this to happen and which ‘binds’ or ties the actions together – writes up the procedure, so to speak – as much as it prescribes action and performs work at each single site, is the instructions.

First, the instructions provide the *rules of the game*. These rules are for the participants. They provide all the information participants get about the experiment. In that respect, the instructions are very much like the rules for a board game – and they are just as tedious to read. They define players’ goals and roles, detail the sequence of ‘rounds’, and explain the implications of possible decisions and strategies. For example:

You will now play a game with monetary stakes. The rules of the game are as follows.

The game is played by two players: player A and player B. Each player must choose between two possible actions. Player A chooses between actions ‘Left’ and ‘Right’. Player B chooses whether he or she wants a six-sided die to be rolled (action *Roll*) or not (action “Don’t roll”). [the instructions then explain how each player’s payoff will be determined] … The game will consist of six identical rounds. At the beginning of a round, one player B is asked to enter the room in which there are six player As…. Player B is then placed in front of player As and remains silent. Then, player B is allowed to talk for no longer than 20 seconds …. (Experiment C)

Second, the instructions are used by the experimenters and their colleagues interested in the study, in that the *design of the experiment* is written in, and together with, the instructions. Writing the instructions is very concretely about arranging the experiment itself – and not just because it happens together with the programming of the interface that materializes the experiments for participants. Indeed, when they write the instructions, experimenters make several choices that can influence the quality of the results.

One choice the experimenters make when writing instructions is how to organize the sequence of events in the experiment, thereby setting the timing and choreography of the economic lab. This is key to how the experiment proceeds; the instructions enable the procedure to be adhered to and organize how each part is played as well as the order in which the whole game unfolds. Because of this, writing instructions involves a lot of deliberations on design details. As one interviewee explains, these deliberations address what is needed for the purpose of the experiments [1], but also what is practical to carry out with the participants [2]:

The design of the experiment is a lot of work. … The basic design was the same: We just re-do the same experiment with the strategy methods. [1] But then, there is also, how many periods do we need, is it essential to have ten periods, is it essential to have twenty periods in the end, could we do with just five, just one … We were thinking about doing the strategy method from the start. [2] And then, we realized that, well, there are reasons why we won’t do … First of all because it’s easier to explain. Because you explain one thing at a time, and then you play it. And then you also see some different things in the different parts. (Experimenter 3)

The instructions also organize and materialize the incentives that are the crux of economic experiments. Incentives set the participants in motions and are often understood as a means to control their actions and motivations. The instructions materialize the incentives as ‘payoffs’ in so-called ‘experimental currency units’ (ECU) and make clear how they will be earned and paid:

You will earn money in the experiment. How much you earn depends on the decisions you make, as well as on the decisions made by other subjects.

…

After the last period ends, your payoffs in ECU are converted to {the local currency} at the stated exchange rate. Your earnings in{the local currency} will be paid in cash as you exit the lab. (Experiment B)

In fact, the instructions mostly consist in explaining what the payoffs are and how the decisions of the subject and the decisions of other participants will affect them, including numerical examples of decision-trajectories.

The elaboration and calibration of the arithmetic of payoffs largely take place when writing the instructions. Like the sequencing, the payoff structure – which also, in fact, structures the game – is written in a back-and-forth process to translate the design ideas in numbers while remaining understandable for participants. For instance, for one experiment, there were long discussions on whether to use intervals or rounded numbers in the questions for participants.

[T]he strategy method here is: They choose 13 levels, but the average of the two others could be anything, from 0 to 60, it could be 29, or 23.5 … So exactly, what you are asking about, is it ‘what do you give if everybody is giving between 20 and 25’, or is it ‘between 17.5 and 22.5’? And we figured out that the things that were easier to say were if it’s rounded to the closest five, and then, we don’t specify it any further … that’s kind of something … Where [that] was a bit back and forth: what is the easiest to explain, how do we avoid misunderstandings … But there is a lot of discussions on details like that when we design the experiment. And we can spend an hour on a detail like that. (Experimenter 3)

Another experimenter explained that ‘there are very long debates on every aspect. How many points do we give, what is the extension rule? … All the parameters, if you want, result from a discussion’, adding that ‘most of the time, it is not an exact science, it’s an art’ (Experimenter 6).

A non-economist would probably not be able to read anything more from the instructions than a sequence of decisions to make and the associated stakes. However, reading the instructions, an experimental economist should be able to retrace the experiment and the logic behind its design. The relative standardization of economic games means that the expert reader will likely be able to pick up the patterns in the game and thus relate the experiment to specific economic questions or theories (for instance, public good games would be associated with questions on cooperation). Thus, instructions make economists accountable for their design choices in front of their absent colleagues.

Third, besides arranging the experiment (that is, the game and the design), instructions also contribute to enacting *the laboratory as a distinct site*. When entering an economic lab, specific rules apply. In fact, more than a specific material apparatus and organization, it is this set of rules and norms that defines the laboratory of economics – what we have called above ‘the laws of the lab’. Again, this is a key part of the procedural elements of the experiment, as these laws prescribe very concretely how to move, act and interact inside the lab. They are spelled out in the instructions, usually in the first paragraphs. This underlines the fundamental role of instructions: Without instructions, there is no economic laboratory, only a room with cubicles and computers.

The rules are simple but strict. Non-compliance can result in exclusion, as a set of instructions states unequivocally:

It is crucial that you understand and obey the rules of this experiment. Violation of these rules might result in an exclusion from the experiment and all payments. (Experiment C).

As the following example shows, the laws of the lab usually include:

- a restriction on free communication with other participants^10^ [1]
- a guarantee of anonymity [2], and a related specification that experimenters will not be able to link decisions to individuals [2′]
- an invitation to raise one’s hand when needing assistance [3]

The results from this experiment will be used in a research project. Therefore, it is important that you follow certain rules. [1] It is important that *you do not talk or in other ways communicate* with any of the other participants during the experiment. Please turn off mobile phones, and use only pre-opened software on the computer. [2] In the experiment, there will be *full anonymity*, which means that no other participants in this room will know which decisions you in particular make during the experiment. [2′] In addition, *it is not possible to track the decisions made during the experiments back to individuals*. You will be notified when the experiment starts, and when you can start entering your answers on the computer in front of you. [3] If you have any questions during the experiment, *please raise your hand, and an experimenter will come to you and answer your question in private*. (Experiment A, emphasis added)

Together, the rules of the game, the laws of the lab, and the design of the experiment build the laboratory as a distinct space-time, write the choreography of the experiment, and frame the interactions and agencies of participants (e.g. but not necessarily, isolated, anonymous individuals who are fully informed and act independently and rationally, that is, strategically to maximize their preferences). As we have noted above, this choreography simultaneously shapes how the experiment and the participants in it shall proceed. The instructions enact the procedure. All of this is put together by crafting a specific genre of text-device. What we will now turn to is how this genre is about crafting instructions that adhere to the discipline of economics.

### Writing good instructions: Enforcing clarity, truthfulness and abstraction

The instructions as a whole create a setup governed by economic norms and somewhat controlled by the experimenters. They simultaneously isolate the participants in the experiment from outside and non-controlled influences, and set new norms, interactions and rhythms that apply to the laboratory space. The instructions are not alone in performing this isolation and motion-setting. As Böhme (2016) shows, they are assisted by the physical environment of the lab, the supervision of experimenters and the computer interface and infrastructure. But these elements are, in large part, centred on making the instructions work: They reinforce them by building in anonymity, making sure they are understood and facilitating the logistics of the game.

The instructions work not only by conveying meaning, but also through writing conventions and techniques that ensure that the text moves participants according to economic norms. Economic experimenters write instructions so that they are clear, truthful and abstract.

By writing *clear* instructions, experimenters try to ensure that participants will understand them. As interviewees explain, if participants understand the instructions, and hence the rules of the game, they are more likely to play the game that experimenters think they are playing. Interviewees explain that the clearer the instructions, the more control experimenters have over what happens in the lab. In that sense, clarity is both a practical and an epistemic concern. On the practical side, clarity is a requirement to be able to make sense of what happened in the lab and of the resulting data – the occasional aberrant data point can be overlooked as resulting from one misunderstanding, but if there’s a suspicion that too many participants misunderstood the instructions, then the data will be too noisy to be used. But this has an epistemic dimension as well, as it relates to what is considered good practice in experimental economics. Several interviewees stress that one should try control the design instead of tampering with the data afterwards: ‘The idea is to control everything we can … *ex ante*. Because afterwards, controlling *ex post*, we can always do it, but it’s not as good’, Experimenter 6 explains. Experimenter 5 concurs, saying that ‘if you control the lab, the art or the science of lab experiments, you can reduce noise in your data, but you never eliminate it. I think that you *are not* allowed to select from your data afterwards’. As a device for experimental control, clarity also serves to demonstrate to readers that the experiment was a compelling performance, that is, that the behaviours and causalities observed were not random but indeed emerged from the situation performed in the experiment.

Clarity stands out in our interviews as a central concern during the writing of the instructions. While experimenters ‘never know if [the participants] really understand the instructions’ (Experimenter 3), they use different techniques to make their instructions clear. When drafting, some of their design choices are informed by the need to avoid misconceptions. They test the draft among themselves, working ‘to come to agreement that this is clear’, in the words of Experimenter 4, who proceeds thus:

When I have finished a first draft of the instructions, I will send it out to the others with screenshots and they will be very critical. And they know what the study is, right, so … So if it’s unclear to them, then it will be unclear to the students, right.

Before the experiment, experimenters carry out pilots with both colleagues and participants; based on direct feedback and on the data they obtain, they can assess whether instructions were understood well enough, and re-write them if needed. During the experiment, they use control questions to check participants’ understanding. These are typically questions about the instructions themselves. Participants can only start playing the game once they have answered those questions correctly.

For instance, Experimenter 5 explained how they realized that their instructions were not clear enough after a pilot test:

So, first of all, we can tell that people are completely lost, you see, in front of the others. They don’t know which players they are and what they should say, why and how. And then, we had a short debriefing. And there, we saw that … We were not clear enough.

Following the test, they thus reworked the instructions:

The experiment being rather complex, we thought that maybe, you see, we should make it easier for them. We should reduce uncertainty. We should just tell them, here is what happens now, here is what will happen afterwards. … You see, it’s small stuff, for instance … We said: Here ‘you play the role of player A/B’, and in the end: ‘You play the role of player …’, because, at the end of the instructions, they did not remember. … So, you see, these are small things. These are not big manipulations, like we’d need to change everything. No, it’s small stuff. But, sometimes, if you put too much faith in, I don’t know, concentration, cognitive capacities, I don’t know, people’s patience … well … you fail. (Experimenter 5)

Another experimenter pointed out the usefulness of printed instructions and control questions as ways to both maximize and measure understanding:

[B]ut here, they had the printed instructions, so they could go back to the instructions at any point during the questions. … Usually they don’t do that much because they are caught up in the task, but especially during control questions it’s good, because it’s a way for them to reactivate what they have heard and to put it to the test. So we do that, usually. … For my experiments, I always do, there is always a screen, and I record how many mistakes they make, what mistakes they make, how many times they enter answers before they are done with the task, and that is kind of a proxy for their understanding. Usually, those who have clicked 20 times before passing, we see aberrant things in their data, because they have not really understood …. (Experimenter 6)

By writing *truthful* instructions, experimenters translate the norm of non-deception into written form. Concretely, this implies that whatever information they give to participants has to be true. The purpose of truthfulness is that participants know all they need to know (in the context of the game) about the decisions they will make and their implications, and that they are aware that they do not need additional information. In this way, participants have no reason to suspect hidden motives. They can trust the experimenters. Concretely, experimenters write in the instructions that everyone has the same instructions, and often read them aloud at the beginning of the session to demonstrate it. If participants trust the experimenters and believe the instructions provide all the information they need, it is supposed that they are less likely to let other considerations play into their decisions. Thus, truthfulness minimizes concerns on the experimenters’ side that participants are motivated by things that are not accounted for or controlled. If the game is, so to speak, self-sufficient, it is more likely that the incentives are what drives participants’ behaviours. So, like clarity, truthfulness enables experimenters to have more control over what participants do. It does not imply that participants know everything: For instance, experimenters typically do not tell participants the purpose of the experiments, lest it influence their strategies.

Last, writing *abstract* instructions is another technique experimenters use to cut out potential interferences. To produce abstraction, players are called ‘player A’ and ‘player B’; if they play in groups, the groups will be labelled ‘X’ and ‘Z’. Like clarity and truthfulness, abstraction is a literary technique to enforce experimental control and to discipline the experimental subjects. It is not always used to the same extent: Some experiments include realistic elements, because this is part of what they want to test. For instance, in one of the experiments in our corpus, participants were given a choice to donate money to the Red Cross. However, aside from this reference to the outer world, the instructions remained very abstract, avoiding, for example, references to colours.

Good instructions are indeed expected to speak for themselves and not trigger any interpretation or association that could interfere with the incentives and sully the story told by the game. These two quotes from interviews exemplify different aspects of this reasoning:

The reason why we want to have it abstract is to avoid that the respondents put too much of their … – They come into the labs with lots of thoughts about the real world, or their thoughts about what they think that *we* want them to do, or … I think that’s the reason why we try and make abstract. And then, the argument is that, if we, in this abstract situation, where there actually is no firms, and … And they don’t know the people they cooperate with, if we *there* can find cooperation and so on, then, we expect that that would also be the case in a real world setting – but – maybe in a real world setting we would see much more of it. (Experimenter 2)

We work on the assumption that incentives incite. That we have designed a task such that they will do what is best for them, given their preferences. For instance, in the dictator’s game, well, the task was designed because, if I’m selfish I will keep everything, and if I want to give to someone I don’t know, then I give to someone I don’t know. And, in fact, I *pay* for that. (Experimenter 6)

Our analysis of the instructions shows the objectivity of economic experiments to hinge on writing style and writing skills. The experimental setup is constructed in a collective and iterative writing process that moves back and forth across the literature, the laboratory, the research hypotheses and the programming of the software. We have shown that this writing process is shaped by the demands of experimental work: The instructions have to perform in the lab. But it is also influenced by a second destination of the instructions, because they are to be appended to an academic paper. Let us now follow the instructions out of the lab, and into the confrontation between the experiments and the discipline of economics that takes place with referees during the publishing process.

### The hand of the referee: How the discipline shapes experiments through peer-review

The product of an economic experiment is publication in a relevant journal, that is, in a journal recognized by the economics discipline – the higher ranked, the better. Ahead of that, papers will often be published as working papers. Indeed, according to our interviewees, the review process can be quite long – some mentioned papers that took several years to publish, which they did not consider unusual.

Experimental economics papers are usually quite long, commonly up to 20 to 30 pages, with lengthy appendixes. They describe the experiments, but in strikingly less abstract terms than the instructions. Writing the paper, economists add flesh and, quite literally, colour to the experimental games, linking them to the economic questions that interests them. The groups named ‘X’ and ‘Z’ in the lab become ‘red’ and ‘blue’ in the paper, and the abstract games played in the lab are made into stories about political corruption, innovation or cooperation within firms. At first glance, the peculiar text of the instructions may seem to have disappeared, but it is actually still part of the paper. The instructions are attached in the appendix, along with extra graphs, data and screenshots of the lab computers. They are thus tied to the paper and move with it through the submission and publication process.

The appendix includes supporting material that does not fit neatly in the paper’s narrative but can vouch for its soundness. One experimenter described the relations between the main text and the appendix as such:

Here we back up our arguments, ‘as shown in appendix …’. So we show that we’re not just claiming things, that we actually have done analyses that support our claims. So there’s some robustness checks and stuff here. (Experimenter 2)

Putting the instructions in the appendix is definitely not hiding them away. To the contrary, it allows them to play a crucial part in the peer-review process. In our interviews, experimenters frequently linked the fact that they included instructions in the appendix to their own practice as referees for other papers. From their accounts, instructions are among the first things that referees look at in their work to assess whether they ‘can believe the result’ (Experimenter 6). For example, they will read them to ‘see if in the instructions, they had suggested a behaviour to the subjects’ (Experimenter 6). Part of the referees’ job is to check whether the instructions adequately implement a controlled economic situation that conforms to disciplinary norms.

Referees come up frequently in our interviews, so much so that we could say that the hand of the referee intervenes in the very writing of the instructions. Indeed, in writing their instructions and designing their experiments, economic experimenters anticipate potential referees’ comments. This suggests that the collective expectations and norms of economics are sufficiently entrenched for them to shape the concrete wording of economists’ experimental devices.

The careful consideration of what referees might think also signals their power over the whole process. According to Experimenter 6:

The way it works with referees’ reports, is that they have the power and we do not have any. And the referees have all the power, so, even if their hypotheses are very eccentric, well … they are right until we have proved them wrong. And that’s the way it is. And … it’s not very good to reply to a referee saying ‘well it’s up to you to prove it’. Then, you are rejected for sure.

The shadow of the referee is there throughout the writing process; in fact, it is (quite literally) enacted by the authors themselves, as the same interviewee explains:

Usually, we act as our own referee. We keep saying: if I were a referee, what would I say here? And we do that with the co-author. I play the referee, and I try to demolish your paper. I say: ‘Well, no, so this, this does not work at all, *because* …. And, oh no, wait, you have to find an answer to everything. It’s quite stressful! (Experimenter 6)

Through this anticipation and role-playing of the referees, the discipline and its validation procedures are involved in writing experimental instructions. Instruction-writing is done with a back-and-forth checking of the literature and the standards, showing how the discipline – embodied in the published literature and the journal process – takes part in experimental design. Standards can also protect from difficult referees. As Experimenter 3 explains, for smaller details,

if there is kind of a standard in the literature, it’s better to follow the standard in the literature than to do it on your own … We’re doing the conventional thing but the referee thinks it’s a bad idea, but anyway, we’re doing it conventionally, so the referee can’t really complain.

The collective rules that define the experimental practices of economics are, so to speak, incorporated in experiments through their very concrete influence on the writing of the texts that enact the experimental setting.

### Building the discipline

The referees’ influence must be seen in the light of the disciplinary organization of economics. The discipline is structured around a well-established hierarchy of journals, with a very selective ‘top-5’ often considered to define the mainstream – or, as some economists recently put it in harsher terms, a ‘tyranny of the top-5’ (Heckman and Moktan, 2019). This hierarchy is maintained by national and disciplinary rankings. For instance, Norway’s ‘level 2’ represents the top-15, while in France a ‘CNRS 1’ journal is better than a ‘CNRS 2’. Strikingly, when asked about the norms or good practices of experimental economics, our interviewees often replied not just in terms of how to produce a sound experiment, but also – sometimes first – in terms of what could be published in a good economic journal.

None of the papers we discussed in our interviews were published in top-5 journals. Nonetheless, the issue of where to submit comes out as a concern in all the interviews. The overall strategy appears to be to submit ‘to the highest-ranked journal in that [sic] we think there would be a chance for acceptance’ (Experimenter 1). Most of the papers we discussed were recent, so still at the submission or revision stage. These were submitted to the *Revue Economique* (‘not far from 90% rejected articles, so that’s significant’, according to Experimenter 7), the *American Political Science Review* (a political science journal, and ‘it’s one of the top-2’ in political science, Experimenter 4 said), *Management Science* (‘because you always need good publications’, Experimenter 5), and the *European Review of Agricultural Economics* (‘less recognized than the *Economic Journal*’ where the same group had published previously, but they ‘wanted to get it published pretty quickly, so [they] didn’t aim very high’, Experimenter 8). We also discussed two published papers. One was published in the multi-disciplinary journal *Research Policy*, after several rejections ‘including the worst rejection of my life’ (Experimenter 6). The last one was published in a special issue of *PNAS*, a very prestigious journal despite not being an economics journal. This was ‘a very unusual process’ (Experimenter 1), but it does count as a good publication. As one of the authors explained, ‘We got a cake! That is – in the economics department, there is a cake list. … actually, since it’s not an economics journal, it’s not formally on the cake list, but … – on regular intervals, there is a cake event to honour the cake publications’ (Experimenter 3).

The clear hierarchy and demarcation of what counts as a good economics journal helps understand the influence of referees in the writing of the paper and of the instructions. Because it matters so much where a paper gets accepted, referees act as gatekeepers assessing whether they can believe the results presented in submitted papers and whether the experimental situation conformed with economic procedures. According to our interviewees’ accounts of their own practices as referees, they rely on checking the instructions. The instructions provide enough information to turn referees into ‘virtual witnesses’ (Shapin and Schaffer, 1985) able to judge whether the lab could be trusted to address economic questions. This process maintains the textbook version of experimental economics which provides standards and shared practices according to which referees judge.

Since the instructions are part of the paper in addition to being part of the experiments, they are written for a double audience: the experimental subjects and the referees. Interestingly, the process of drafting and testing instructions when designing experiments appears very similar to refereeing work. As Experimenter 6 puts it, experimenters ‘act as [their] own referees’. The same set of skills and expertise come into play when designing one’s own experiment and reviewing another’s, and these skills largely pertain to the writing and appraising of instructions.

Good instructions need to fit a journal, referees’ standards and expectations, and the economic story told in the paper, in addition to being part of a working experimental setup. Importantly, once the instructions are made part of the paper, they serve another purpose than they did in the lab. After orchestrating the performance in the lab, the instructions attached in the appendix testify that it conformed with the objectivity requirements of economics. The ability to write a text that can perform these two tasks is, we argue, the core skill of experimental economists, a skill that is honed by designing experiments collectively and by reviewing papers.

## Conclusions: Accomplishing objectivity

This paper analyses how experimental economics is made by following experimental instructions, as they move between the labs and the literature of economics. Our ‘text-author ensemble’ method enabled us to reconstruct the procedures involved in doing experimental economics, and to foreground instruction-texts as material-semiotic devices central to the work of experimental economists and to the performance of experimental economic results. The instructions, we argue, perform two crucial operations: They build the lab and the experimental setup itself (because they spell out the rules of the game and the experimental design), and they inscribe them within the discipline of economics (by way of peer review, shared norms and by being a text that allows other economists to retrace the experiment). Written texts then appear as constitutive of the materiality, practices and discipline of economics. By being simultaneously semiotic *and* material, things in the world *and* carriers of meaning, experimental instructions constitute the sites and the procedures of experimental economics and build the discipline one journal article at a time. As they move, they link the lab and the discipline, the world in the experiment and the world of economics. Thus, this text-device is not simply rhetorical or textual, it is also crucially a material and moving entity; it is an experimental instrument.

As a result of their remarkable roles, the instruction-texts prompt us to reconsider the relations between texts and objectivity. Instructions are central to the production of the objectivity of experimental economics, but as opposed to the narrativizing that Morgan (2012) has shown to be an epistemic practice that makes sense of models and links them to the world outside, instructions cannot be reduced to the stories and meaning they convey. Their epistemic value stems from their very construction, materiality and circulation *as texts.* Yet, instructions cannot be understood as inscriptions (Latour, 1995; Latour and Woolgar, 1986) either; they do not serve to translate experimental objects (or, in the case of economics, subjects) from the lab to the paper, but to create the very lab and to make subjects move inside it. They are texts, but they are texts that are simultaneously experimental instruments.

We have shown that instructions work by combining procedural, material and rhetorical functions. By being written so that participants can follow them thoroughly and unequivocally, they serve to produce interpretable data and to reduce noise, and therefore to minimize the need for *ex post* control. They are thus devices to ensure experimental control, which help identify causalities and make sure that observations relate to the question investigated. In the submitted and eventually published articles, they take part in demonstrating that the experiments were compelling performances and not collections of random decisions made according to unknown, uncontrolled and unaccounted for factors. Throughout the article, we have also pointed at how the instructions take part in ordering the experimental work. It would be tempting to say that instructions ensure that the experiments follow and comply with procedure (Porter, 1995). However, this does not capture their significance. In contrast with protocols in natural science experiments, the instructions are integral to experimental work and actively take part in producing the procedure. Texts in experimental economics are crucial to the production of objectivity not just in the form of literary technologies that enable virtual witnessing, but also as instruments that build the very experimental setting and procedure.

But texts need to be written, so this objectivity is not accomplished only by what the instruction-texts say. It is also produced in the very act of writing them and in the way instructions move, first among colleagues, then in the lab, then into the literature. The crafting of such text-devices involves a lot of skill and expert judgment, as our interview quotes suggest. It is ‘an art, not an exact science’ to quote Experimenter 6, mastered by moving back and forth between the literature, the computer programme, the lab and the text, through discussions and tests among colleagues, and through learning when to stop refining the text and settling on maybe imperfect, but good enough instructions. Reminiscent of the way bureaucrats describe note-writing skills as a crucial part of their expertise (Mangset and Asdal, 2019), the quality of economic experiments seems to hinge on a collectively maintained ability to write good instructions.

The objectivity of economic experiments is thus accomplished by a combination of impersonality – that is, detaching the procedure from individual subjectivity – and personal writing skill. This is comparable with bureaucracy: In order to be a good and successful bureaucrat, impersonality is not enough. You also need the skill to write a good submission that can move within the bureaucratic system. Hence, writing is a distinct form of expertise that is crucial to being a good bureaucrat and to uphold bureaucracy (Mangset and Asdal, 2019). The same is true for the social sciences: Objectivity ultimately hinges on the skill of writing – a skill that is linked to a role and a profession, but is nevertheless intertwined with the individual, with trained judgment and with the art of combining the rhetorical and the epistemic.

Experimental economics is a case in point. Instructions are written so as to produce a form of mechanical objectivity (Porter, 1995) in the lab; they are supposed to suppress subjectivity, biases and uncontrolled influences. Yet, the objectivity of the experimental setup requires a great deal of qualitative literary work, and skill in judging and writing a good text. Instructions must be clear, abstract enough, truthful and relevant to economic analysis. These skills are trained by reviewing other economists’ papers, and they are disciplined by unwritten procedures and choreographies of collective work. In that sense, and perhaps contrary to expectations, laboratory experimental economics remains closer to literary work and model-based abstraction than to laboratory sciences.

The textual, in combination with the material, accomplishes objectivity. In the case of experimental economics, this combination is embodied in the text-device of the written instructions that perform economics in distinct ways at each stage in the knowledge production process. It is the very tight connection of the textual (the narrative) and the material device which is key to how economics is made to perform *as* economics. That is why these need to be traced in combination.

## Notes

1. We thank an anonymous reviewer for suggesting this formulation.

2. Doganova and Eyquem-Renault (2009) similarly analyse business models as devices that not only combine narratives and calculations, but also circulate and, by circulating, build a network for the innovation they represent.

3. http://leem.umontpellier.fr/index.php?page=labos=fr, retrieved 25/10/2019.

4. One interviewee offered to try the experiment with one of the authors before the interview. This was a mock-experiment, as it was improvised and no payoff was given in the end, but it provided an experience of what it is like to act as a subject and to face the instructions. The same author had previously taken part in a psychology experiment.

5. Economic experiments are not always so abstract, even in the lab – one of our interviews was about an experiment that involved recreating a grocery store – but games involving abstract tasks were dominant in our sample.

6. Interviewees explained that economics students tended to try to perform well – that is, to behave according to what they learned in their textbooks or lectures – while psychology students expected to be lied to, meaning that both would tend to adopt unnatural behaviours in experiments.

7. As Bardsley et al. (2010: 36) note, method in economics is often defined as ‘a set of relatively uncontroversial rules of good practice, internal to scientific discipline, of which established scientists have a tacit understanding and into which novices are induced’. Citing Hands (2001), they contrast it with ‘methodology’ as an abstract topic for discussion and reflection in specialized journals.

8. Despite their moral connotation, the terms ‘deception’, ‘lie’ or ‘honesty’ should here be understood in a very technical way. Lying, or not lying, are two distinct techniques to induce behaviours in subjects, and ensure that these behaviours have epistemic value.

9. The software package is free, but publications from experiments using z-Tree must cite a 2007 article presenting the software. As of March 2021, this paper had been cited over 5400 times according to Crossref. https://citations.springer.com/item?doi=10.1007/s10683-006-9159-4

10. Communication is not always completely banned, and can sometimes be the object of study, but it is always subjected to specific rules.
